# Comparative Biophysical Analysis of Healthy and Inflamed Intestinal Membrane Models Using Langmuir Monolayers

**DOI:** 10.1021/acs.jpcb.6c01059

**Published:** 2026-07-07

**Authors:** Michalina Zaborowska-Mazurkiewicz

**Affiliations:** University of Warsaw, Faculty of Chemistry, Pasteura 1 02093, Warsaw, Poland

## Abstract

Intestinal inflammation, including Crohn’s disease, represents a chronic condition that increases the risk of colon cancer and involves complications from long-term pharmacotherapy. Since drugs must pass through lipid bilayers to reach their targets, understanding the surface properties of biological membranes is crucial. In this study, the Langmuir technique and Brewster angle microscopy (BAM) were employed to characterize simplified models of healthy and inflamed intestinal cell membranes. Both the outer and inner leaflets of the lipid bilayer were modeled to investigate how inflammation-induced changes in lipid composition, fatty acid saturation, and pH affect membrane integrity. The results demonstrate that inflamed models exhibit increased fluidity, reduced molecular packing, and lower collapse pressures compared to healthy ones. Notably, the study reveals a significant reduction in the differentiation between the surface properties of the outer and inner monolayers in the inflamed state, indicating a loss of membrane asymmetry. These findings provide novel physicochemical insights into inflammation-driven membrane remodeling, potentially supporting the design of targeted therapies with improved absorption in pathological states.

## Graphical abstract

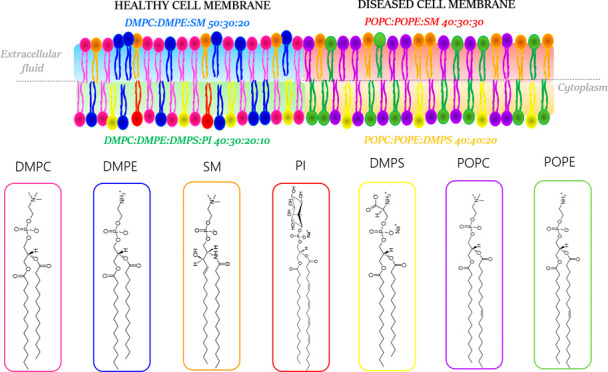

## 1 Introduction

Crohn’s disease (CD) is a serious type of inflammatory bowel disease (IBD).^1^ A significant increase in people suffering from this disease has been observed in highly developed and developing countries, especially since the beginning of the twenty-first century.^2,3^ Crohn’s disease dates back to 1932 when Burrill Bernard Crohn diagnosed a group of patients with ileitis, although symptoms of severe enteritis were first described in 1904 by the Polish surgeon Antoni Leśniowski.^4^ Nowadays, we are still unable to give a specific reason for this disease, usually pointing out disorders of the immune system, lifestyle, and stress or inheritance. Crohn’s disease is most often associated with problems related to the gastrointestinal tract (GI) (but it comes in different phenotypes^1^), its main symptoms include profuse and bloody diarrhea, abdominal pain, vomiting, and inflammation of the entire body. The main attention is focused on changes in the intestines - significant thickening of the intestinal walls, ulcers, and fissures in the intestinal wall are observed.^5^ These are macro changes, visible and noticeable to the body of a sick person, however (like many diseases) their pathogenesis begins in the cells. Epithelial cells (mainly enterocytes) are connected by several proteins, e.g. occludins, claudins, cadherins, and catenins (called tight junctions, TJ),^6,7^ create a barrier that allows only what is needed for the proper functioning of the body and protects against toxins and harmful microorganisms. Disturbance of the functioning of the above-mentioned proteins, and thus changes in the lipid bilayers constituting the barrier, lead to increased permeability of the intestinal wall, which causes inflammation in the body. JAK/STAT (Janus kinases/Signal Transducer and Activator of Transcription) pathway is responsible for the transmission of the signal from cytokine anchored into the cell membrane to the cell interior and the activation of transcription in the cell nucleus. This in turn activates inflammation and the immune response. Another factor leading to intestinal stenosis occurring with CD and thus to its obstruction is the progressive increase in cytokines and transforming growth factor (TGF-β) associated with inflammation.^5^

Intestinal epithelium membranes are characterized by the abundance of sphingomyelin (SM) and phosphatidylcholines (PC).^8,9^ Other lipids present in the membranes of these cells are phosphatidylethanolamines (PE) and phosphatidylserines (PS), as well as phosphatidylinositol (PI).^10^ The membrane composition is as follows: PC = 40%, PE = 23%, SM = 23%, PS = 8%, PI = 4% and 2% of other lipids.^11^ In the event of inflammation, changes in the lipidomic profile of lipid membranes include an increase in the ceramide component (mainly SM) at the expense of phosphocholines.^12^ A shift toward a lower PC-to-PE ratio has been reported, together with a pronounced reduction in phosphatidylinositol-derived lipids (for PS lipids, no difference was observed between the membranes of healthy cells and those affected by Crohn’s disease).^10^ Particular attention should be paid to changes in the amount of phosphatidylinositol-derived lipids,^10^ because, as many biological studies revealed, their reduction leads to increased membrane permeability and changes the cell membrane curvature,^13^ which is conducive to the development of Crohn’s disease.^14^

Alterations in cholesterol content have been reported in inflamed intestinal epithelial membranes, contributing to increased permeability and impaired barrier function.^15^ Although cholesterol strongly affects membrane fluidity and packing, it was omitted from our models to focus on the effects of phospholipid headgroup composition and fatty acid saturation, allowing clearer interpretation of lipid–lipid interactions under healthy and inflammatory conditions.

In addition to headgroup remodeling, elongation of the acyl chains in specific phospholipids of intestinal epithelial membranes (e.g., phosphatidylcholine and phosphatidylethanolamine) has been reported in inflammatory conditions such as Crohn’s disease, with chain length increasing by several carbons on average^10,16^ Such elongation can influence membrane fluidity and packing, contributing to increased permeability of the small intestinal epithelium - the so-called “leaky gut” - which facilitates the translocation of toxins and microorganisms into the bloodstream and may exacerbate inflammatory responses. Inflammatory conditions within cells are also characterized by a lower-than-physiological extracellular pH (pH = 7.4), typically around 5.4 ^17^. These reported lipidomic alterations reflect global changes in membrane composition and do not resolve leaflet-specific lipid distributions, which remain incompletely characterized under inflammatory conditions.

The above-described layers can be presented as synthetic complex lipid models allowing for a detailed study of intermolecular lipid interactions between individual components in the layers and for a multidirectional assessment of the surface properties of healthy and inflamed intestinal epithelial cell membranes. Until now, the focus was more often on cancer conditions^18^ than on inflammation and phosphatidylcholine models of the intestinal mucosa and its interaction with mucin. We also used a simple gut model a few years ago: DMPC/DMPE/DMPS 4:4:2 as the first barrier for the absorption of drugs used against high blood cholesterol levels. When analyzing the cited lipidograms,^8–10,15^ the DMPC:DMPE:SM 5:3:2 / DMPC:DMPE:DMPS:SAPI 4:3:2:1 (pH = 7.4) system can be used as a simple model of healthy intestinal epithelium, and the POPC:POPE:SM 4:3:3 / POPC:POPE:DMPS 4:4:2 (pH = 5.4) system, corresponding to the outer and inner monolayers of the lipid bilayer, can be used as an inflammatory model. These asymmetric monolayer compositions represent simplified experimental models designed to capture the major lipidomic trends associated with inflammation, rather than exact leaflet-specific lipid distributions.^19,20^ In the healthy monolayer models, fully saturated DM-lipids (DMPC, DMPE) were selected despite their relatively low abundance in natural membranes, because their short, saturated acyl chains lack cis double bonds and therefore exhibit a higher packing capability upon compression, reflecting cooperative packing and mechanical characteristics of healthy intestinal membranes. In the inflammatory models, PO-lipids (POPC, POPE) were used to mimic elongation and partial unsaturation of acyl chains observed in inflamed membranes.^21^ This strategy allows the models to represent both headgroup remodeling and chain-length effects, which together influence membrane fluidity, molecular packing, and permeability, rather than to induce an artificially rigid or fluid phase state.

One of the most invaluable methods for creating biomimetic lipid layers is the Langmuir method.^22–25^ For complex lipid models, such as the lipid membranes of intestinal epithelial cells mentioned earlier, it is an ideal technique for creating complex (multicomponent) compositions at the air/water interface, which closely resembles the aqueous environment found in cells. In this context, Langmuir monolayers serve as a well-established reductionist model that enables high sensitivity to compositional changes at the membrane interface, while intentionally omitting bilayer-specific effects such as interleaflet coupling and cholesterol-induced condensation. The Langmuir–Blodgett method is a biophysical technique that, in addition to enabling the study of molecular packing in the membrane (surface pressure—area per molecule isotherms) and investigation of their elastic properties (the dependence of the compressibility coefficient on surface pressure), also allows for calculations of the forces and interactions between components in the layer (*A* ^Exc^, *% A*). It can also assess the miscibility of components (Δ*G* ^M^) and detect the potential formation of irreversible aggregates based on thermodynamic parameters (while recording compression and decompression cycles of the layers).^26^ An important parameter emphasizing the preferential nature of the structures formed by given lipids is the critical packing parameter (CPP), described many times in the literature.^27^ Moreover, it allows the evaluation of the electrical properties of the layer by measuring the membrane’s surface potential.^25,28–31^ Additionally, compressing the layer to a specific pressure enables the assessment of layer stability under conditions similar to those in real cell membranes. Another method used for creating layers at the interface is the assessment of the layer’s morphological changes during compression, visualized through Brewster’s angle microscopy (BAM).^32–34^ For complex systems (such as three-and four-component mixtures) like those selected for this study, the above parameters provide valuable, complementary information. The understanding of the properties of the monolayers building the membranes of healthy and affected cells will support further research on the development of treatments for Crohn’s disease by enabling the testing of new therapeutics and assessing the effectiveness of drugs used in CD while minimizing side effects on healthy cell membranes.

## 2 Experimental Section

### 2.1 Materials

The following lipid set was used in the study: 1,2-dimyristoyl-*sn*-glycero-3-phosphocholine (DMPC), 1,2-dimyristoyl-*sn*-glycero-3-phosphoethanolamine (DMPE), 1-stearoyl-2-arachidonoyl-*sn*-glycero-3-phospho-(1′-myo-inositol-5′-phosphate) (ammonium salt) (SAPI), Sphingomyelin (SM, extract mainly with16-carbon chains) 1,2-dimyristoyl-*sn*-glycero-3-phospho-l-serine (sodium salt) (DMPS), 1-palmitoyl-2-oleoyl-glycero-3-phosphocholine (POPC), 1-palmitoyl-2-oleoyl-glycero-3-phosphoethanolamine (POPE). Lipids are characterized by high purity (>99%) and are soluble in organic solvents such as chloroform/methanol (4:1, v/v) (at a concentration of approximately 0.5 mg/mL). All lipids and solvents are commercially available and were purchased from Merck (Darmstadt, Germany). Four mixed lipid models were prepared to characterize the outer and inner leaflets of the biological membrane in both healthy (outer: DMPC/DMPE/SM 5:3:2, inner: DMPC/DMPE/DMPS/SAPI 4:3:2:1) and inflamed states (outer: POPC/POPE/SM 4:3:3, inner: POPC/POPE/DMPS 4:4:2).

Britton–Robinson buffer (BR) containing boric acids (H_3_BO_3_), acetic acid (CH_3_COOH), and phosphoric acid (H_3_PO_4_) titrated to pH 5.4 and 7.4 at a fixed ionic strength of 0.01 M was used as a subphase in the studies. The buffer was prepared using Milli-Q double-distilled water with a resistivity of 18.2 MΩ·cm (Millipore).

### 2.2 Methods

The monolayers at the interface were formed using the Langmuir method and specialized equipment. The main component is a Langmuir trough with movable hydrophilic barriers, which compress the lipid layer at the air/water interface at a rate of 10 mm/min (Langmuir trough area 243 cm^2^). The surface pressure (±0.1 mN/m) relative to the decreasing area per molecule is measured using a Wilhelmy plate suspended on a Wilhelmy balance. Surface pressure (π) is the difference in surface tension of the pure subphase (γ_0_) and the monolayer (γ).^25^ Surface pressure stability over time (π–*t*) was measured by compressing the layers to a target surface pressure of 30 mN/m. Once the target pressure was reached, the barriers were stopped, and the surface area was held constant. This specific value was selected because it corresponds to the internal lateral pressure of native cell membranes, thus enabling the evaluation of monolayer stability under biologically relevant conditions. The relaxation of the surface pressure was then monitored for at least 3 h. The measurements were performed at a temperature of 22 ± 1 °C. This temperature was chosen to ensure the stability of the monolayers during long-term experiments, as well as to allow for a direct comparison with a broad range of existing literature on Langmuir lipid models.^35,36^ All Langmuir isotherms were recorded in at least ten independent replicates. The results showed high reproducibility, with surface pressure deviations within ±0.5 mN/m and area per molecule deviations within ± 1 Å^2^/molec.. The curves presented in the figures are representative isotherms.

By recording the surface pressure–area per molecule (π–*A*) isotherm, it is possible to calculate the reciprocal of the compressibility modulus (*C*_s_^*–*1^) using the formula (eq 1).^24,37^ This parameter provides information on the elastic properties of the obtained layers, according to the following ranges: (0–12.5) mN/m for the gas phase (G), (12.5–100) mN/m for the liquid-expanded phase (LE), (100 – 250) mN/m for the liquid-condensed phase (LC), and above 250 mN/m for the solid phase (S).

1 $$C_{s}^{-1}=-A{\left(\frac{d\pi }{dA}\right)}_{T}$$

Additional information can be obtained by analyzing the positions (relative to the surface pressure) of the minima and maxima of the compressibility modulus. The minima, depending on whether they reach zero or not, can be attributed to either partial collapse of the layer or reorganization of its structure, respectively. The maximum indicates the phase in which the obtained layer is most organized.

Moreover, by examining the recorded isotherms, it is possible to estimate a range of thermodynamic properties of the obtained layers, including the excess area (*A* ^Exc^), excess Gibbs mixing enthalpy (Δ*G* ^Exc^) and total Gibbs mixing energy (Δ*G* ^M^).^26,38,39^

2 $$A_{1..N}^{id}=\sum_{1}^{N}A_{i}X_{i}$$

3 $$A^{Exc}=A_{1...N}-A_{1...N}^{id}$$

4 $$\Delta G^{Exc}=N_{A}\int_{0}^{\pi }A^{Exc}d\pi$$

5 $$\Delta G^{M}=\Delta G^{Exc}+\Delta G^{id}$$

6 $$\Delta G^{id}=RT\sum_{1}^{N}X_{i}{\ln X}_{i}$$

The excess area (*A* ^Exc^) values approximate the interactions occurring between the components in mixed layers by comparing them to the ideal interactions in single-component layers. A negative excess area value indicates more attractive or less repulsive interactions, while a positive excess area value indicates more repulsive or less attractive interactions. This parameter is calculated based on the theoretical area value, called the ideal area (*A* ^id^), which is calculated using eq 2. Here, *A*_*i*_ represents the area per molecule for a pure component at a given surface pressure, *X*_*i*_ is the molar fraction of the given component, and the area value for the mixed layer (*A*_1···*N*_) is read at a given surface pressure.^40^ An additional excess parameter is Δ*G* ^Exc^ (eq 4), which refers to the excess free enthalpy in the system resulting from the mixing of components (again compared to layers where the components mix perfectly). It should be noted that while Δ*G* ^Exc^ strictly refers to excess free enthalpy, in the investigated monolayer system (operating at the air/water interface under constant lateral surface pressure) Δ*G* ^Exc^ can be considered equivalent to the excess Helmholtz free energy (Δ*F* ^Exc^). This parameter helps us understand the effects (increase or decrease in free energy) that occur in the mixed system under study. Furthermore, we can determine the total mixing energy (Δ*G* ^M^) (eqs 5 and 6), which represents the total thermodynamic force driving the mixing process and is a key parameter in understanding the stability of mixed monolayers.

Data that provide insight into the thermodynamics of the layers can be also obtained by recording the compression and expansion cycles of the layer and analyzing the hysteresis.^39,41,42^ Such experiments enable the assessment of lipid layer reorganization processes and may indicate the occurrence of domain formation or aggregation phenomena, as well as their reversible or irreversible character, as inferred from the hysteresis behavior. The thermodynamic parameters related to hysteresis are described by the equations below

$$W_{comp\;or/and\;exp}=N_{A}\int_{1mN/m}^{30mN/m}A_{comp\;or/and\;exp}d\pi$$ 7

8 $$W^{hys}=W_{\exp}-W_{comp}$$

9 $${\left[\Delta S_{\pi }^{hys}=R\ln\frac{A_{\exp}}{A_{comp}}\right]}_{\pi }$$

10 $$\Delta S^{hys}=\sum_{\pi }\Delta S_{\pi }^{hys}$$

11 $$\Delta H^{hys}=W^{hys}+T\Delta S^{hys}$$

the work of hysteresis (*W* ^hys^), the configurational entropy of hysteresis (Δ*S* ^hys^), and the enthalpy of hysteresis (Δ*H* ^hys^) can be calculated based on the above equations.^39,41,42^ *W*_comp or/and exp_ (which represents the mechanical work performed during compression/expansion) is calculated based on the values of the area in a given range of surface pressures for the compression–expansion curve (*A*_comp_ */A*_exp_) with Avogadro’s number (where *N*_*A*_ is equal to 6.022 10^23^ mol^–1^) (eq 7). Compression refers to the decrease of the monolayer area, while expansion corresponds to its increase. The work of hysteresis (*W* ^hys^) can then be accurately calculated using eq 8. The values are mostly calculated starting from 1 mN/m to avoid disturbances in the surface pressure measurements at the very beginning. Then, using above values of the surface area per molecule (*A*_comp_/*A*_exp_), Δ*S* ^hys^ values (eqs 9 and 10) can be obtained with constant *R* (the universal gas constant 8.314 J·mol^–1^·K^–1^).

Using the Langmuir technique, the surface potential (Δ*V*) of the layers can be also measured. This parameter provides additional information about the packing of molecules during layer compression and changes in their orientation. To determine the changes in potential, the apparatus is modified by incorporating a platinum counter electrode (placed under the surface of the subphase) and a Kelvin probe (positioned just above the surface of the subphase) to record changes in surface potential (±1 mV). Δ*V* is defined as the potential difference between the subphase and the covered subphase. According to the above, the change in potential is related to the apparent dipole moment (μ_*a*_), the permittivity in vacuum (ε_0_ = 8.8542 · 10^–12^ F m^–1^), the permittivity of the monolayer (ε), the value of which is unknown.^29^ Due to this fact, the apparent dipole moment (μ_*a*_) (eq 13) is used. From this experiment, two curves can be determined: the dependence of the potential change (eq 12) on the area per molecule (Δ*V* vs *A*), and the dependence of the apparent dipole moment (μ_*a*_ vs *A*), since the monolayer at the air/water interface is treated as a set of dipoles that can contribute to its polarization.

However, for monolayers containing ionizable lipid species, the total measured surface potential (Δ*V*) can be theoretically described by extending the Helmholtz equation to account for the double-layer potential (ψ_0_) resulting from lipid dissociation^43^

12 $$\Delta V=\frac{\mu_{a}}{{\varepsilon \cdot \varepsilon }_{0}\cdot A}+\psi^{0}$$

13 $$\mu_{a}=\frac{\mu_{a}}{\varepsilon }=\Delta V\cdot A\cdot \varepsilon_{0}$$

in this study, the explicit contribution of ψ_0_ was not calculated. Because all experiments were strictly conducted on a subphase with a relatively high and constant ionic strength (0.01 M BR buffer), the electrostatic charges of the dissociated headgroups were significantly screened. Consequently, the variations in Δ*V* between the investigated systems can be primarily attributed to the structural reorganization and changes in the effective orientation of the lipid dipoles (μ_*a*_).

A different experiment is Brewster angle microscopy, which allows determining the morphology of the layer with a resolution of up to 2 μm. The measurement is performed simultaneously with the recording of isotherms using the UltraBAM attachment using the Nanofilm_ep3 setup (Accurion, Germany).

## 3 Results and Discussion

To study interactions in mixed layers, we modeled healthy (pH = 7.4) and inflamed (pH = 5.4) small intestine epithelial cell membranes composed of different lipids (Figure 1). We examined the surface properties of the component monolayers. Figure S1 shows the surface pressure - area per molecule isotherms, with key parameters summarized in Table S1. Supporting Materials contains a brief, referenced description of the methods for characterizing surface properties.

The DMPC:DMPE:SM 5:3:2 and DMPC:DMPE:DMPS:SAPI 4:3:2:1 compositions under pH = 7.4 conditions can serve as representative models of the healthy intestinal epithelial membrane. Emphasizing the elongation and increased degree of unsaturation, as well as the rising proportion of SM and the absence of SAPI lipids as well as lower pH value equal to 5.4,^8,10^ the POPC:POPE:SM 4:3:3 and POPC:POPE:DMPS 4:4:2 models - corresponding to the outer and inner leaflets of the lipid bilayer, respectively—are appropriate for modeling the inflamed state. The π – *A* isotherms of mixed layers reflecting the monolayers of small intestine epithelial cell membranes are presented in the figures below.

**Figure 1:**
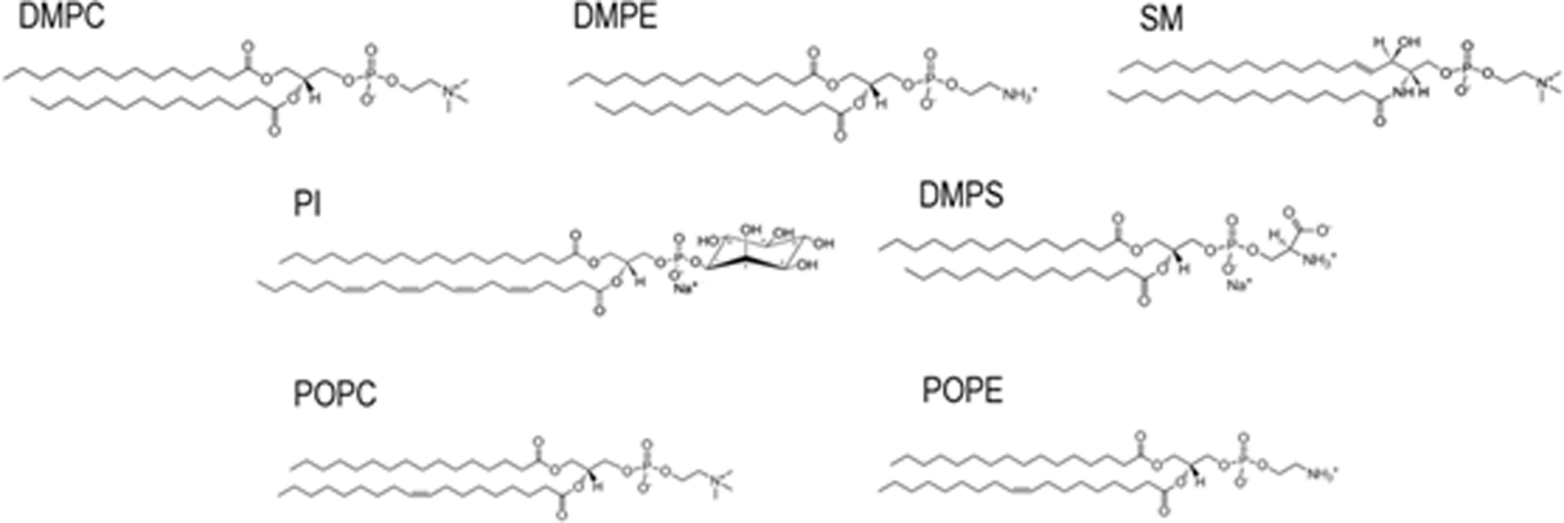
Chemical structures of lipids used in selected models.

In the ternary mixture DMPC/DMPE/SM 5:3:2, characteristic of the outer layer of a healthy biological membrane, a relatively tight packing of the hydrophilic heads is observed at the air/water interface. This is evident at surface pressures of π = 10 mN/m (*A* = 63 Å^2^/molecule) (Figure 2A and Table 1). At lower surface pressures (π < 20 mN/m), the monolayer exhibits a liquid-expanded phase, and at π = 30 mN/m, the area per molecule is equal to 40 Å^2^/molecule. However, as the structure reorganizes, it transitions into a liquid-condensed phase, where lipid–lipid interactions occur through both polar and hydrophobic forces. Negative values of the excess area and the mixing free energy (Figure 3A,B) signifying the presence of attractive or weakly repulsive forces, along with a higher degree of layer condensation (Figure 3, blue). Moreover, after the subsequent phase transition, better miscibility of components in the layer is observed (Δ*G* ^M^ ∼ (−2.5 – −4.5) kJ/mol). It is also important to recognize that, according to the theory of preferential structure formation, DMPC and SM, with critical packing parameter (CPP) values ranging from 0.5 to 0.75, tend to form planar layers, while DMPE tends to form cone-shaped structures.^44^ Therefore, it is unlikely that the monolayer is homogeneous at higher surface pressure values, as reflected in the Brewster angle microscopy images (Figure 2C). The reorganization of the monolayer is reflected in the appearance of “flower-like” domains, which result from the morphology of the DMPE and SM lipids. Both lipids form stable monolayers with a high degree of packing, thanks to the presence of many hydrogen bonds. As compression of the ternary layer continues, the domains begin to fuse at a surface pressure of 40 mN/m, with a predominance of LC regions while LE areas are still visible, indicating LE–LC coexistence. This is in good agreement with the calculated compression modulus value at given surface pressure (∼90 mN/m).

**Figure 2:**
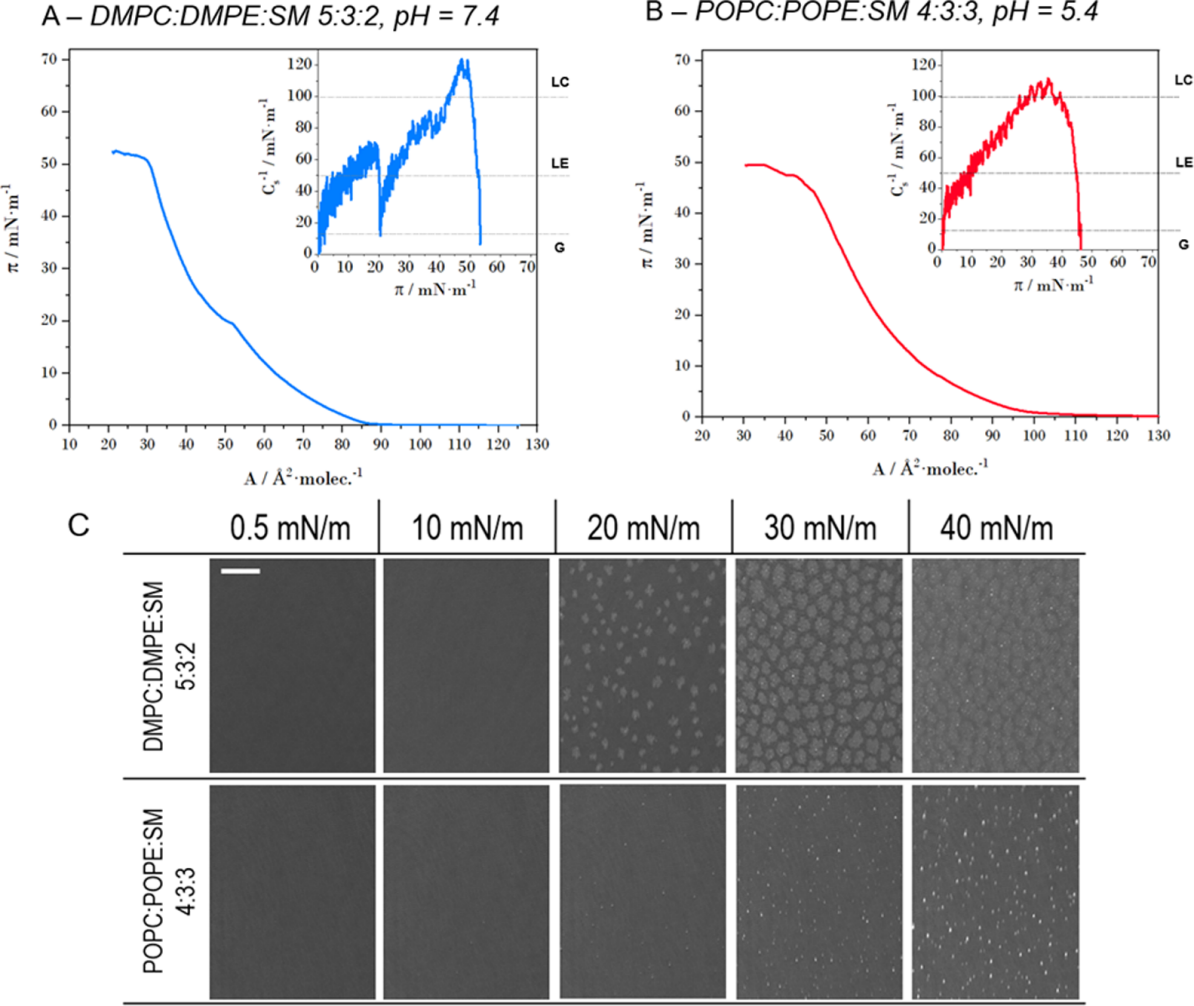
Surface pressure – area per molecule isotherms for healthy (A) and inflamed (B) monolayers showing the compositions of outer leaflets of lipid membranes. Insets: dependence of the compressibility modulus on the surface pressure (*T* = 22 ± 1 °C). (C) Images obtained by Brewster angle microscopy showing the morphology of both monolayers. Scale bar is 100 μm (*T* = 22 ± 1 °C).

**Table 1:** Parameters Characterizing the Langmuir Single- and Multi-Components Monolayers^a^ ^b^

subphase	lipid model	*A*_π=10 mM m_^–1^/Å^2^ molec.^–1^	*A*_π=30 mM m_^–1^/Å^2^ molec.^–1^	*C*_s_^–1^_max_/mN m^–1^	Δ*V*_max_/V	μ_a, max_/D
BR buffer, pH = 7.4	DMPC:DMPE:SM 5:3:2	62.8 ± 0.2	40.0 ± 0.2	112 ± 10	0.546 ± 0.006	0.619 ± 0.002 (90.0 ± 0.5)
	DMPC:DMPE:DMPS:SAPI 4:3:2:1	71.1 ± 0.5	45.6 ± 0.3	96 ± 8.0	0.420 ± 0.005	0.592 ± 0.002 (114.2 ± 0.4)
BR buffer, pH = 5.4	POPC:POPE:SM 4:3:3	74.3 ± 0.4	58.5 ± 0.8	110 ± 8.0	0.338 ± 0.005	0.564 ± 0.005 (98.3 ± 0.2)
	POPC:POPE:DMPS4:4:2	74.2 ± 0.1	55.1 ± 0.9	96 ± 6.0	0.364 ± 0.005	0.545 ± 0.005 (100.1 ± 0.7)

a Below μ_a, max_ the values of the area per molecule (Å^2^ molec.^–1^) at which the maximum was reached are shown.

b Values are presented as representative mean ± standard deviation (*n* ≥ 10).

**Figure 3:**
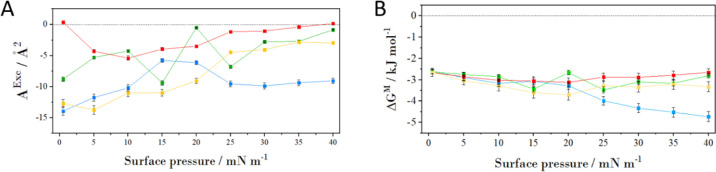
Thermodynamic parameters: excess surface area (A), total mixing energy (B) as a function of surface pressure indicating the quality of interactions in DMPC:DMPE:SM 5:3:2, pH = 7.4 (blue), DMPC:DMPE:DMPS:SAPI 4:3:2:1, pH = 7.4 (green), POPC:POPE:SM 4:3:3, pH = 5.4 (red) and POPC:POPE:DMPS 4:4:2, pH = 5.4 (yellow) layers.

When comparing the mixed layer POPC:POPE:SM 4:3:3 with the DMPC:DMPE:SM 5:3:2 layer discussed above, the presence of lipids with double bonds results in a more loosely packed structure of the layer, consistent with literature data.^45^ Additionally, CPP values for both POPC and POPE indicate truncated cones.^46^ However, this change does not significantly affect the elastic properties of the layer, indicating a strong influence of sphingomyelin (*C*_*s*_^*–*1^_max_ for healthy = 112 mN/m vs *C*_s_^*–*1^_max_ for inflamed = 110 mN/m). It is evident that sphingomyelin interacts strongly with the two unsaturated lipids, and no partial collapse is observed for POPC or POPE (compared to the inner leaflets interactions described below). The complete collapse of the monolayer occurs at a surface pressure and area per molecule of 50 mN/m and 35 Å^2^/molecule, respectively (Figure 2B). This suggests strong interactions of sphingomyelin with unsaturated chains (as supported by literature^47^) as demonstrated in the case of SM-DOPC, and also in studies showing interactions of SM-POPC and their preferential formation of lipid rafts.^48,49^ On the other hand, the values for excess area within the layer indicate the presence of weakly attractive forces between the components over the entire range of surface pressures (Figure 3, red). These forces, along with the increase in surface pressure, arise from the bent chains of oleic acids present in POPC and POPE molecules and the strengthening of hydrogen and hydrophobic interactions between nonpolar parts in lipid molecules. This phenomenon can be also attributed to the presence of two types of alcohol residues in the polar heads.^25^ The primary distinction between these lipids lies in the size of their polar heads, which directly influences their hydration levels. PC lipids, possessing larger polar heads, are less hydrated compared to PE lipids (here we can distinguish the formation of strong hydrogen bonds between NH_3_^+^ and PO_4_^–^ groups).^50–52^ The dominance of unsaturated phosphatidyl lipids over sphingomyelin prevents the formation of domains in the layer’s morphology, with only bright aggregates-like structures observed at high surface pressures (Figure 2C).

In the inner monolayer of the healthy membrane model (DMPC:DMPE:DMPS:SAPI 4:3:2:1), formed at the air/water interface, the coexistence of liquid-expanded and liquid-condensed phases (LE–LC) represents an optimal organization, balancing membrane fluidity and stability, with a maximum compression modulus of *C*_s_^–1^_max_ = 96 ± 8 mN/m (Figure 4A and Table 1). Notably, the multiphasic properties and the distinct shape of the surface pressure–area per molecule isotherm directly originate from the presence of these mixed phases in the monolayer. The negatively charged DMPS stabilizes the condensed regions via electrostatic interactions, while the polyunsaturated PI increases monolayer fluidity, resulting in a physiologically relevant mixed-phase monolayer (Figure S1C and Table S1). The area per molecule is larger than in the healthy outer model; at a surface pressure of 10 mN/m, the difference is approximately 10 Å^2^/molecule. After reorganization occurring around 22 mN/m, which further manifests the multiphasic nature of the compression process driven by phase separation, this difference decreases to 5 Å^2^/molecule at a surface pressure of 30 mN/m. The attractive or weakly repulsive interactions between the components in the layer, confirmed by values of *A* ^Exc^ < 0 (Figure 3A), are also visible through small aggregates at 10 mN/m (Figure 4C). In the region of reorganization, small domains or crystallites are formed, which are visible in Brewster angle microscopy images. These morphological features captured by BAM provide direct, visual evidence that the mixed-phase character of the monolayer is responsible for the observed multiphasic properties of the isotherm. These domains either grow or are surrounded by other domains similar to those observed for the DMPC:DMPE:SM layer. From studies by Horswell et al.,^52,53^ we also know that DMPE has a higher affinity and, therefore, interacts more strongly with PS lipids than with PC, hence the high heterogeneity of the layer. BAM images clearly show that these domains do not completely merge into a homogeneous structure, further supporting the previous hypothesis regarding the imperfect miscibility of the components. Additionally, the sudden increase in Δ*G* ^M^ values at high surface pressures indicates a low degree of miscibility of components in the layer (Figure 3B), which leads to the partial collapse of the layer at approximately 50 mN/m (Figure 4A). Considering the surface collapse pressures of the individual components in this layer, we can assume that it concerns either DMPC (Figure S1A) or SAPI (Figure S1C), which again confirms the strong DMPE-SM interactions.

**Figure 4:**
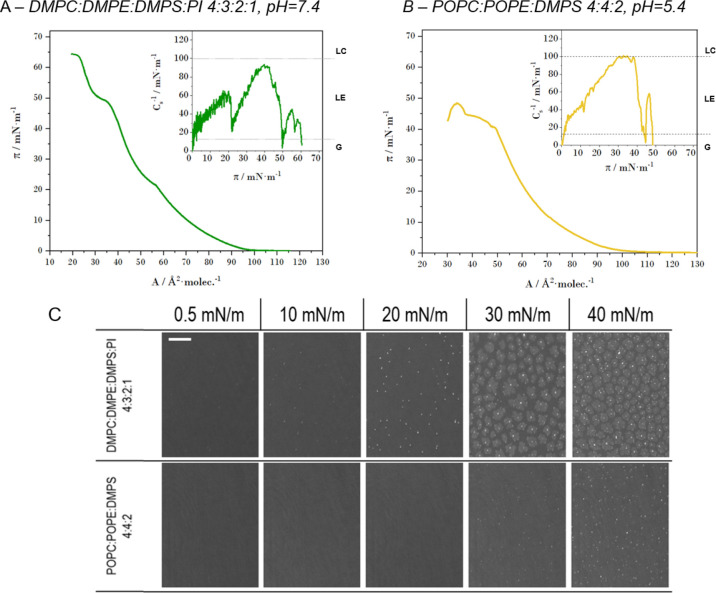
Surface pressure – area per molecule isotherms for healthy (A) and inflamed (B) monolayers showing the compositions of inner leaflets of lipid membranes. Insets: dependence of the compressibility modulus on the surface pressure (*T* = 22 ± 1 °C). (C) Images obtained by Brewster angle microscopy showing the morphology of individual monolayers. Scale bar is 100 μm (*T* = 22 ± 1 °C).

Upon analyzing the properties of the model inner monolayer (POPC:POPE:DMPS 4:4:2), which characterizes inflammation, very similar values for area per molecule and compressibility modulus are observed when compared to the outer layer (POPC:POPE:SM 4:3:3) (Figure 2B and Table 1). In both cases, no phase transitions are noted, indicating the absence of any detected reorganization after the ″lift-off″ point at 100 Å^2^/molecule. However, when comparing the shape of this curve with that of a healthy membrane (Figure 4B vs 4A), the difference is significant. The lipids within the inflamed membrane are loosely packed, and forces resembling repulsive or weakly attractive interactions are present between the components, in contrast to the interactions observed in perfectly miscible single-component layers.^54^ As the degree of layer compression increases, there is a corresponding increase in the proportion of repulsive forces and a decrease in the extent of layer condensation. Initially, Brewster’s microscopy images do not show any specific layer morphology, which perfectly corresponds to the excess parameter values and the conclusions derived from them. At a surface pressure of 40 mN/m, aggregates form, which are visible in BAM images (Figure 4C), whereas no such aggregates are observed at 30 mN/m, corresponding to the physiological properties of real cell membranes.

The above discussion of the findings is further supported by surface potential (and apparent dipole moment) isotherms plotted as a function of the area per molecule. The DMPC:DMPE:SM monolayer exhibits a markedly higher degree of apparent dipole alignment (as well as Δ*V*), reflecting a more uniform orientation of molecular dipoles - particularly their polar head groups – concerning the interface. The hydrophilic headgroups are immersed in the aqueous subphase, while the hydrophobic tails are directed toward the air, forming a well-organized monolayer. The elevated apparent dipole moment observed for this composition suggests a predominant orientation of dipoles nearly perpendicular to the air/water interface. Additionally, the presence of strongly polar moieties, such as carbonyl and phosphate groups, at the interface further enhances the dipole contribution. The reduced fluidity of the layer implies a more condensed and less dynamic molecular packing, characteristic of rigid, tightly ordered membrane domains. This rapid increase in Δ*V* indicates the onset of domain formation, even though such domains remain undetectable in the Brewster angle microscopy (BAM) images (Figure 2C). It is worth noting that this method is particularly sensitive to the initial stages of monolayer formation, as it can detect even subtle lipid reorientations when treated as dipoles. In contrast, the BAM technique is a macroscopic imaging method that visualizes domains formed by aggregates of lipids (with image dimensions of 430 × 800 μm).

For the inner, healthy lipid monolayer, the inclusion of serine- and inositol-derived lipids promotes earlier molecular organization (relative to the gas phase) and supports the vertical alignment of the hydrocarbon chains at larger area per molecule values (∼110 Å^2^/molecule) (Figure 5B, green curve). The maximum apparent dipole moment (μ_*a*_), corresponding to the transition from the gas to the liquid-expanded phase, is approximately 0.564 D (Figure 5B, black curve). It should be emphasized, however, that in such multicomponent systems, these maximum values serve as operational and qualitative indicators of collective packing rather than absolute physical constants. Because the local dielectric permittivity (ε) within the polar headgroup region is highly dynamic and changes nonlinearly upon compression, direct quantitative comparisons of absolute maxima across different mixtures remain limited.^43^ To provide a more comprehensive view of this dynamic behavior, Table 1 also includes the specific molecular area values at which these maximum apparent dipole moments were reached. Nevertheless, for comparative purposes at the point of optimal monolayer organization - i.e., at a surface pressure of around 40 mN/m - the change in surface potential reaches 0.420 V, slightly lower than the value observed for the outer monolayer (Table 1).

**Figure 5:**
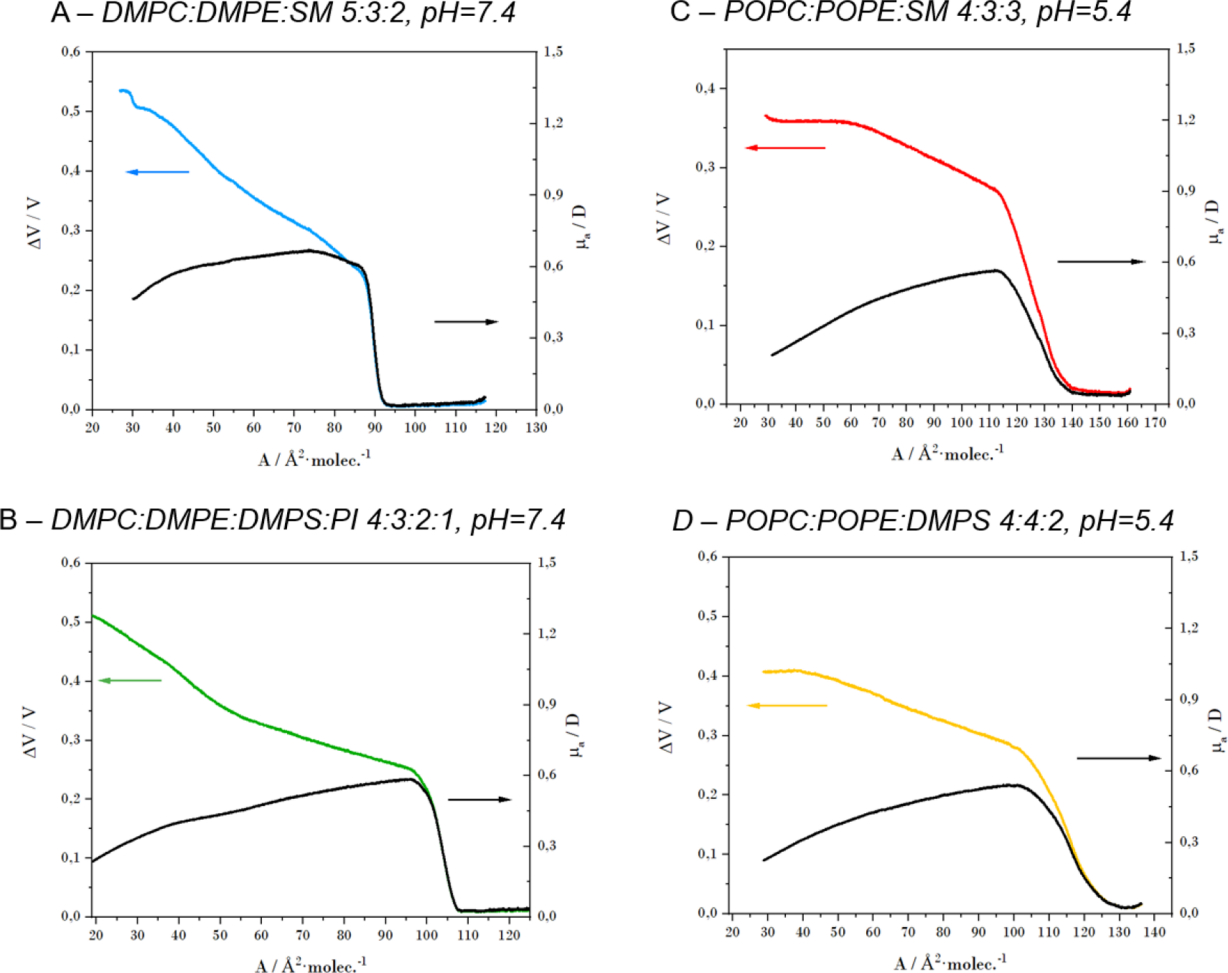
Surface potential (left axis) and apparent dipole moment (right axis) – area per molecule isotherms for healthy (A,B) and inflamed (C,D) monolayers showing the compositions of outer (A,C) and inner (B,D) leaflets of lipid membranes (*T* = 22 ± 1 °C).

For the outer layer mimicking inflamed membranes, the reorganization of apparent dipole moments - associated with potential-forming groups such as C=O - occurs at larger molecular areas (approximately 140 Å^2^/molecule) than the transition from the gas to the liquid-expanded phase which occurs only at a surface of 100 Å^2^/molecule. Carbonyl groups significantly influence the organization of lipid molecules at the air/water interface due to their position just above the subphase surface, while the hydrophilic headgroups are submerged in the subphase and shielded by it.^55^ This phenomenon of the earlier appearance of interactions influencing potential changes may also be due to strong interactions between lipid molecules in the mixture already at the moment of their distribution at the interface, which is probably due to the presence of more hydrophobic nonpolar parts (the presence of double bonds). It should be noted that the increase in potential is more gradual than in the healthy membrane model (compare Figure 4A with 4C, blue and red curves). When analyzing surface potential isotherms and the apparent dipole moment for the inner inflamed membrane model, no significant differences are observed compared to the outer monolayer (Table 1), suggesting a conceptually similar qualitative trend in their maximum macro-dipolar alignment despite their differing corporate compositions.

The POPC:POPE:SM and POPC:POPE:DMPS monolayers, representing lipid compositions associated with inflamed membrane states, exhibit low apparent dipole moment values and a reduced or only slightly changing surface potential (Δ*V*). These characteristics suggest a lack of pronounced dipole reorganization upon monolayer compression, indicating that the dipoles may already be disordered or maintain a relatively constant orientation throughout the process. The increased fluidity and lower degree of molecular ordering within these layers reflect a more dynamic and loosely packed structure. This disorganization may result from the presence of less polar or more flexible lipid species, or dipoles adopting a more parallel orientation relative to the interface, thereby diminishing the net dipole contribution perpendicular to the surface. Inflammatory conditions are frequently associated with such membrane behavior, as the disruption of lipid packing and reduced dipolar response are indicative of compromised structural integrity and altered biophysical membrane properties. These findings support the hypothesis that inflammation correlates with weakened dipole alignment, greater monolayer disorder, and a decline in the overall dipole potential. Moving on to the previously mentioned membrane dynamics, the studies also investigated the reversibility of the formed domains/aggregates, which could be visually observed in the Brewster angle microscopy images (Figures 2C and 4C). For this purpose, compression and decompression cycles were recorded and the thermodynamic parameters described in the Experimental section were determined.

Upon compressing the DMPC:DMPE:SM monolayer to 30 mN/m and subjecting them to decompression (hysteresis cycles), irreversible aggregates are formed, as each subsequent cycle shifts toward lower values of the area per molecule (Figure 6A). From an energetic perspective, this system represents the most thermodynamically favorable state, indicating strong inter- and intramolecular interactions. The negative value of *T*Δ*S* ^hys^ further suggests an entropically favorable phenomenon (Table 2), and additionally confirms the formation of a condensed phase in the layer and thus the loss of freedom of movement of the molecules. Additionally, when the barriers are maintained at a surface pressure of 30 mN/m (with no further compression), and changes in surface pressure are recorded over time, it is evident that the formed domains lack stability (Figure 7). This is evidenced by a slightly decrease in surface pressure for PC:PE:SM healthy monolayer, followed by the establishment of a plateau at approximately 23 mN/m, and one can conclude that the resulting irreversible structures may contribute to the destabilization of the layer. Notably, compression of the DMPC:DMPE:DMPS:SAPI layer to 30 mN/m does not lead to the formation of irreversible structures (Figure 6B), which can be attributed to the nearly perfect miscibility of the components within the layer at 30 mN/m (*A* ^Exc^ ∼ 0) (Figure 3A). Furthermore, it is evident that these interactions positively affect the stability of the monolayer overtime at 30 mN/m, as illustrated in Figure 7.

**Figure 6:**
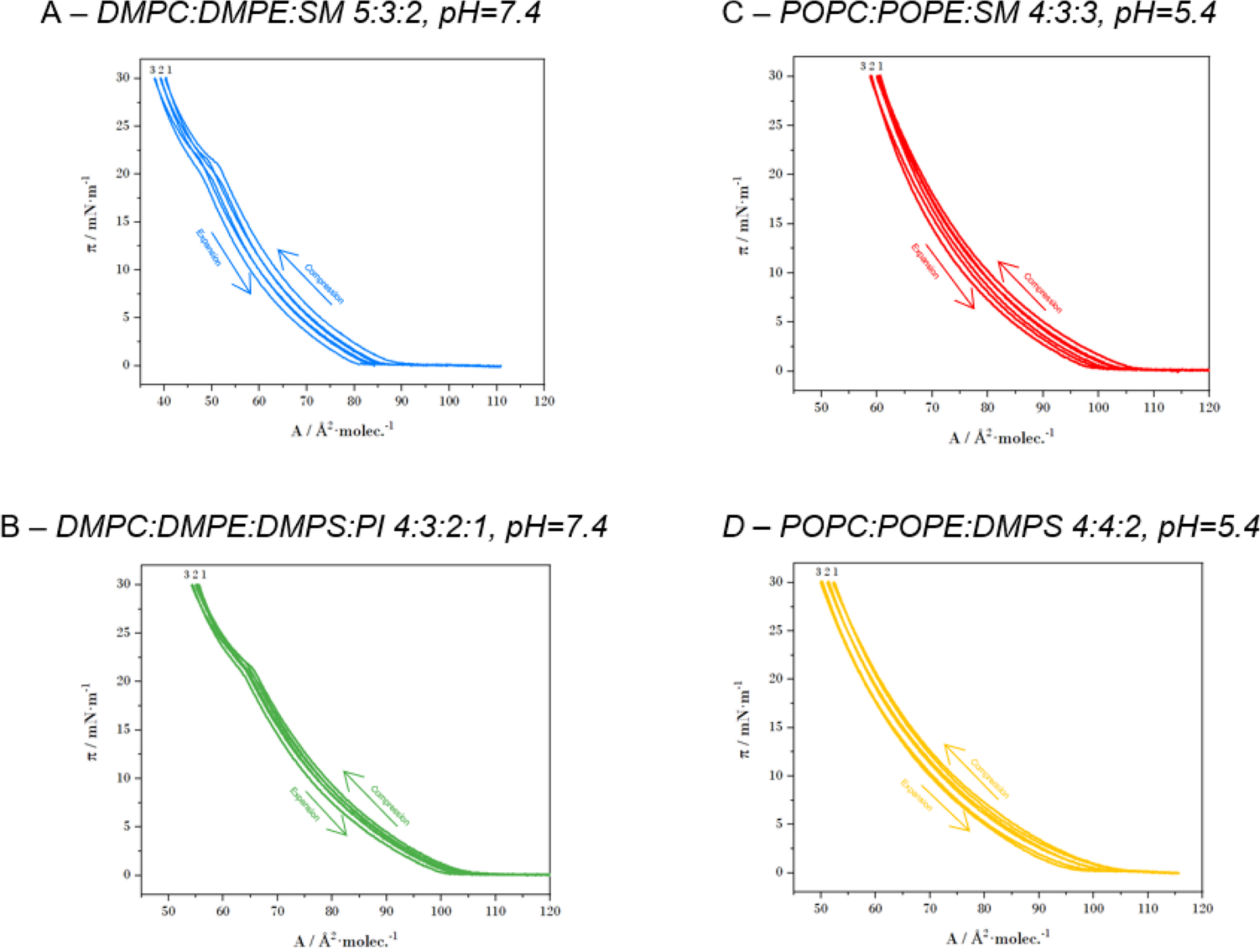
Compression–expansion cycles (hysteresis) for monolayers compressed to 30 mN/m on Britton-Robinson buffer with pH = 7.4 for healthy lipid models (A and B) and pH = 5.4 for inflamed lipid models (C and D) (*T* = 22 ± 1 °C).

**Table 2:** Thermodynamic characteristic parameters for the monolayers calculated on the basis of recorded first hysteresis cycle (eqs 7–11)­^a^

*W*_comp_/kcal·mol^–1^	*W*_exp_/kcal·mol^–1^	*W* ^hys^/kcal mol^–1^	TΔ*S* ^hys^/kcal·K mol^–1^	Δ*H* ^hys^/kcal mol^–1^
DMPC:DMPE:SM 5:3:2				
0.812 ± 0.005	0.722 ± 0.004	–0.091 ± 0.0003	–0.564 ± 0.002	–0.677 ± 0.005
DMPC:DMPE:DMPS:SAPI 4:3:2:1				
0.781 ± 0.004	0.677 ± 0.005	–0.104 ± 0.0002	–0.503 ± 0.007	–0.606 ± 0.005
POPC:POPE:SM 4:3:3				
0.728 ± 0.002	0.673 ± 0.002	–0.055 ± 0.0005	–0.217 ± 0.004	–0.272 ± 0.008
POPC:POPE:DMPS 4:4:2				
0.677 ± 0.001	0.631 ± 0.005	–0.0473 ± 0.005	–0.209 ± 0.003	–0.256 ± 0.004

a Values are presented as representative mean ± standard deviation (*n* ≥ 3). Parameters for the remaining cycles are included in Table S2 in the Supporting Materials.

**Table 3:** Comparative Summaryof Physicochemical and Structural Properties of Healthy and Inflamed Intestinal Membrane Models

parameter	healthy membrane	inflamed membrane
area per molecule (at π = 30 mN/m)	∼40–45 Å^2^	∼55 Å^2^ (more expanded monolayer)
compression modulus	higher values (stable layer)	lower values (increased fluidity)
morphology	uniform lipid domains	small, aggregates-like structuresat higher surface pressure
asymmetry between outer and inner leaflet	present	Reduced
collapse pressure (π_coll_)	>50 mN/m	<50 mN/m (reduced stability)

**Figure 7:**
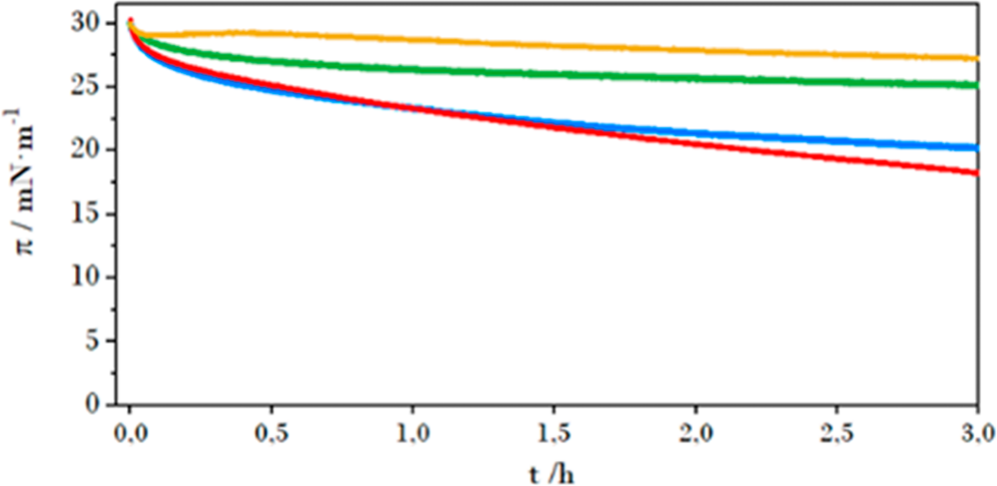
Surface pressure time dependencies indicating the stability of Langmuir monolayers DMPC:DMPE:SM 5:3:2, pH = 7.4 (blue), DMPC:DMPE:DMPS:SAPI 4:3:2:1, pH = 7.4 (green), POPC:POPE:SM 4:3:3, pH = 5.4 (red) and POPC/POPE:DMPS 4:4:2, pH = 5.4 (yellow) previously compressed to a surface pressure of 30 mN/m (*T* = 22 ± 1 °C).

It is not surprising that the introduction of double bonds into the system will change the thermodynamic characteristics of the layers. Again, small hysteresis loops are seen where sphingomyelin is present in the layer. The observed instability at a given surface pressure over time results from the specific nature of the lipids, particularly the presence of double bonds in the fatty acid chains, which are predominant in the mixture. Consequently, it is not surprising that for POPC:POPE:DMPS layer *W* ^hys^ is close to 0, and no hysteresis loops are observed (Figure 6D). Therefore, the presence of a negatively charged lipid in the layer promotes better miscibility of the POPC/POPE components compared to SM. In the case of monolayers modeling inflamed membrane environments, such as POPC:POPE:SM, the less negative values of these parameters point to a lower degree of molecular ordering and increased monolayer fluidity. These findings reflect smaller energetic differences between the compressed and relaxed states, implying that the system exhibits a reduced tendency toward ordered packing. This behavior may be attributed to membrane perturbations typically associated with inflammatory conditions, which disrupt lipid organization and weaken dipolar interactions within the monolayer.

## 4 Conclusions

Inflammation in diseases like Crohn’s or ulcerative colitis can disrupt intestinal barrier function, leading to increased permeability to toxins and bacteria.^56–58^ By utilizing biomimetic lipid monolayers formed via the Langmuir technique paired with Brewster angle microscopy (BAM), this research successfully modeled cell membranes in both healthy and inflamed states. This approach enabled a controlled investigation of how pathological membrane remodeling and alterations in fatty acid saturation affect key physicochemical parameters. Our findings reveal that inflamed membrane models exhibit a significantly larger area per molecule (*A*_π=30 mN/m_ = 58 Å^2^/molecule) and lower collapse pressures (π_coll_ < 50 mN/m) compared to healthy models, indicating a more expanded, fluid, and less mechanically stable structure that reflects the compromised barrier function observed in vivo. Furthermore, a pivotal observation of this study is the reduced differentiation between the surface properties— - including molecular packing, BAM morphology, and electrical properties - of the outer and inner monolayers in the inflamed state, signaling a severe loss of native lipid asymmetry (Table 3). Additionally, while healthy models show uniform lipid domains, compression of the inflamed models leads to the formation of macroscopically heterogeneous, aggregate-like structures at higher surface pressures, which may potentially alter the lateral organization of membrane-associated proteins.

While Langmuir monolayers are simplified models that lack cholesterol, native bilayer complexity, and were studied at 22 °C to ensure optimal BAM resolution, excluding these factors enabled a focused, direct assessment of the individual contributions of phospholipid remodeling and fatty acid saturation. The observed trends - specifically increased fluidization and loss of asymmetry - are expected to be further amplified at physiological temperature (37 °C), confirming the representative nature of the applied system.

In conclusion, this controlled biomimetic platform provides novel physicochemical insights into inflammation-driven membrane alterations. These findings may inform future pharmaceutical and medicinal chemistry studies aimed at designing targeted drug delivery systems with optimized absorption profiles in inflamed epithelial tissues, potentially improving therapeutic efficacy in chronic inflammatory diseases.

## Supplementary Material



## Data Availability

Data are available in the open repository:Data availability Data are available in the open repository: https://doi.org/10.58132/8AR81V.

## Notes

Manuscript—MZaborowska_Mazurkiewicz_revised_22062026 (PDF)
