# Supplementary material for: Comparative Biophysical Analysis of Healthy and Inflamed Intestinal Membrane Models Using Langmuir Monolayers
Source: J Phys Chem B. 2026 Jul 7;130(28):7045–56. doi: 10.1021/acs.jpcb.6c01059 (PMC13383736; doi:10.1021/acs.jpcb.6c01059)
Supplement: Supplementary file 1 [file jp6c01059_si_001.pdf]

Supporting Materials

Comparative biophysical analysis of healthy and inflamed intestinal membrane models using Langmuir monolayers

Autor Michalina Zaborowska-Mazurkiewicz[a]

Affiliation [a]*Faculty of Chemistry, University of Warsaw, Pasteura 1, 02093 Warsaw, Poland*

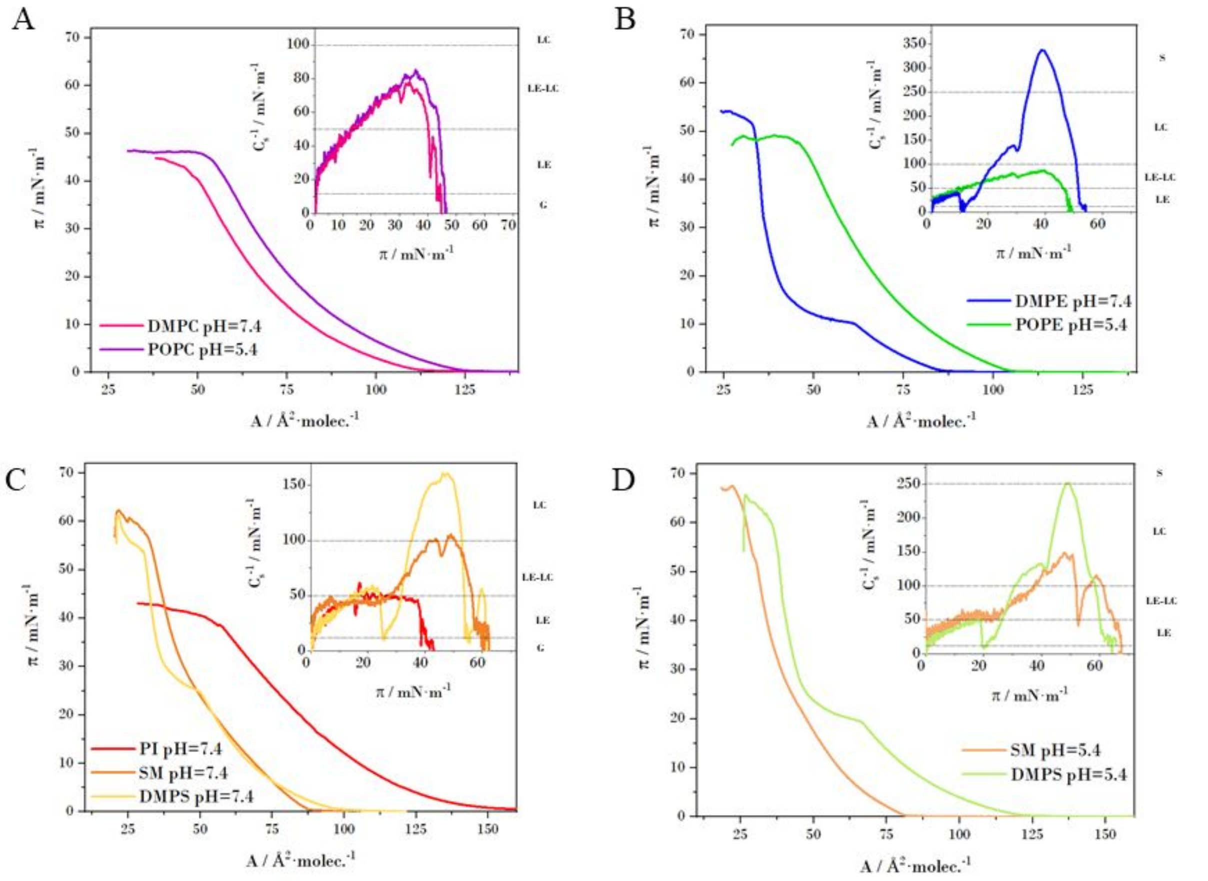

Figure S1 Surface pressure – area per molecule isotherms for single-components layers forming complex models representing healthy and inflamed membranes. Insets: dependence of the compression modulus on the surface pressure (T = 22 ± 1°C).

Table S1 The parameters characterizing the Langmuir single-components monolayers used to form the complex models

Subphase	***$A_{\pi=10\ mM\ m^{-1}}$ / Å² molec.⁻¹***	***$A_{\pi=30\ mM\ m^{-1}}$ / Å² molec.⁻¹***	***$C_s^{-1}{}_{max}$ / mN m⁻¹***
	BR buffer, pH=7.4		
DMPC	81.2±0.2	58.0±0.4	74±9

DMPE	62.2±0.8	36.8±0.8	336±12
SM	67.9±0.2	46.6±0.6	106±8
DMPS	67.2±0.6	38.6±0.5	156±10
PI	103.7±0.2	68.7±0.5	51±6
BR buffer, pH=5.4			
POPC	91.2±0.8	66.4±0.5	88±5
POPE	79.3±0.5	58.2±0.8	87±8
SM	58.7±0.2	39.0±0.3	148±8
DMPS	82.9±0.5	43.6±0.6	251±10

The predominant lipid components in biological membranes are typically phosphatidylcholine (PC) lipids (such as DMPC and POPC) and phosphatidylethanolamine (PE) lipids (such as DMPE and POPE), both of which are zwitterionic. In this study, DMPC and DMPE lipids, as well as DMPS were selected as model lipids because they feature fully saturated 14-carbon hydrocarbon chains without double bonds, which promotes the formation of well-ordered Langmuir monolayers at the air/water interface [1,2]. Although DMPC and DMPE are not abundant in natural cell membranes, their saturated chain structure provides a simplified and reproducible model to study monolayer mechanics. In particular, the packing and elastic properties of these lipids allow us to approximate the mechanical behavior of biological membranes under controlled experimental conditions, making them suitable for investigating membrane elasticity.

The DMPC layer is characterized by a relatively loose packing of polar heads, particularly when compared to other lipids such as DMPE and DMPS. Nevertheless, existing literature supports the stability of DMPC lipid monolayers [3] and “lift-off” point near to 110 Å^2/molec. (*Figure S1A*) [4,5]. The maximum value of the compression modulus suggests that the layer exhibits a liquid-expanded nature. In contrast, DMPE layers are highly ordered, with the area per molecule at a surface pressure of 30 mN/m being approximately 37 Å^2/molec. (*Figure S1B*), which corresponds to the literature data [6]. The compressibility coefficient for DMPE exceeds 330 mN/m, which corresponds to the solid phase of the resulting monolayer.

In contrast to the aforementioned lipids, POPC and POPE are characterized by distinct structural features, including the presence of one palmitic chain, which promotes better ordering compared to myristic acid due to its longer length (similar to the effect observed with in DPPC [7]), and one oleyl chain, which contains a double bond that induces greater disorder and increased

fluidity in the layer (as seen with DOPC [8]). The isotherm recorded for the POPC lipid layer, formed on a Britton-Robinson buffer with pH equal to 5.4, is slightly shifted towards higher values of area per molecule (*Figure S1A*) [9,10]. The obtained compression modulus values equal to 88 mN/m placing this layer in the liquid-expanded phase [11].

The isotherm for the POPE lipid does not exhibit the phase transition observed in DMPE. The POPE layer demonstrates greater "layer relaxation", likely due to the bending of the fatty acid chains caused by the presence of C=C double bonds [10]. This bending weakens the interactions between the polar heads of the phosphatidylethanolamine molecules (NH_3^+ - PO_4^- hydrogen bond). The area per molecule at 10 mN/m in this layer is approximately 20 $Å^2$/particle larger than that in DMPE, and a similar trend is observed at a higher surface pressure of 30 mN/m. Consequently, the compression modulus indicates that the POPE layer is in the liquid-expanded phase (*Figure S1B*) [7].

DMPS lipid layers have been extensively studied in the literature under various conditions, including different subphase compositions [12]. Due to the negative charge on the DMPS molecule, the lipid layers are highly sensitive to these environmental factors. Despite this sensitivity, DMPS layers formed at the subphase with pH near to 7.4 exhibit tight lipid packing (A = 38 $Å^2$/molecule) and a high degree of layer stability, with a maximum compression modulus of 156 mN/m. However, the presence of salt in the BR buffer significantly reduces this value compared to the compression modulus observed for DMPS layers on pure water [13]. For the layer formed on the lower pH subphase (5.4), a solid phase layer is observed where $C_s^{-1}{}_{max}$ equals 250 mN/m (Figure S1D). Furthermore, a characteristic phase transition for DMPS occurs at a surface pressure of ca. 25 and 20 mN/m for higher and lower pH values, respectively (compare *Figure S1C* and *Figure S1D*), whereas on water at room temperature, this transition occurs at a lower surface pressure (~5 mN/m) [14]. The flower-like domains that appear in the above-cited work are very significant in the characterization of monolayers.

Sphingolipids, which are present in both leaflets of biological membranes, were also considered in this study. An extract predominated by (16:0)-sphingomyelin (SM) was used for the analysis. Monolayers of sphingomyelin (16:0), similarly to DMPS, were tested in two environments: one at a higher (7.4, *Figure S1C*) and the other at a lower (5.4, *Figure S1D*) pH. Both layers are characterized by relatively rigid lipid packing, and the maximum C_s^{-1} values place these layers in a liquid-condensed phase, which is consistent with the literature [15–18]. Lower pH conditions form layers with slightly more compact lipid packing, which is

particularly evident at a surface pressure of 30 mN/m, where the area per molecule is 39 Å^2/molecule (compared to almost 47 Å^2/molecule for a pH of 7.4).

Phosphatidylinositol (PI) lipids, extracted from liver and predominated by 18:0 and 20:4 fatty acid chains, form highly fluid layers ($C_s^{-1}{}_{max}$ is equal to 51 mN/m) with loose packing of the polar heads at the air/water interface (*Figure S1C*). At 10 mN/m, the area per molecule is 104 Å^2/molecule, and at 30 mN/m, it is 69 Å^2/molecule. For comparison, for lipid PI (16:0, 18:2) these values are 73.9 and 24.4 Å^2/molecule, respectively, and the value of $C_s^{-1}{}_{max}$ is only 20 mN/m [19]. This loose packing is due to the weak interactions between the phosphatidylinositol residues and the high number of double bonds in the fatty acid chains [20]. Additionally, the research demonstrated the possibility of forming clusters of phosphatidylinositol lipids in contact with an aqueous solution of different size and surface characteristics depending on the surface pressure.

Table S2

Thermodynamic characteristic parameters for the monolayers calculated on the basis of recorded hysteresis cycles.

	$\Delta G_{comp/exp}$ / kcal· mol^{-1}	ΔG_{comp} / kcal· mol^{-1}	ΔG_{exp} / kcal· mol^{-1}	ΔG^{hys} / kcal· mol^{-1}	$T\Delta S^{hys}$ / kcal·K· mol^{-1}	ΔH^{hys} / kcal· mol^{-1}
			DMPC:DMPE:SM 5:3:2			
I cycle	-0.0850±0.0005	0.812±0.005	0.722±0.004	-0.091±0.003	-0.564±0.002	-0.677±0.005
II cycle	-0.0901±0.0006	0.801±0.004	0.703±0.006	-0.099±0.003	-0.661±0.003	-0.760±0.005
III cycle	-0.0878±0.0008	0.790±0.004	0.697±0.002	-0.093±0.002	-0.623±0.003	-0.716±0.005
			DMPC:DMPE:DMPS:PI 4:3:2:1			
I cycle	-0.0990±0.0002	0.781±0.004	0.677±0.005	-0.104±0.002	-0.503±0.007	-0.606±0.005
II cycle	-0.0870±0.0006	0.783±0.003	0.685±0.004	-0.098±0.005	-0.470±0.007	-0.567±0.004
III cycle	-0.0870±0.0004	0.769±0.001	0.680±0.007	-0.089±0.004	-0.425±0.006	-0.514±0.004
			POPC:POPE:SM 4:3:3			
I cycle	-0.0471±0.0002	0.728±0.002	0.673±0.002	-0.055±0.02	-0.217±0.002	-0.272±0.002
II cycle	-0.0751±0.004	0.749±0.005	0.661±0.003	-0.087±0.04	-0.399±0.001	-0.487±0.006
III cycle	-0.0663±0.0009	0.722±0.004	0.648±0.009	-0.074±0.03	-0.314±0.004	-0.388±0.002
			POPC:POPE:DMPS 4:4:2			
I cycle	-0.0396±0.0002	0.677±0.001	0.631±0.005	-0.0473±0.05	-0.209±0.003	-0.256±0.004
II cycle	-0.0404±0.0001	0.695±0.002	0.647±0.002	-0.0472±0.02	-0.201±0.002	-0.248±0.002
III cycle	-0.0564±0.0002	0.696±0.002	0.635±0.002	-0.0611±0.02	-0.301±0.002	-0.362±0.002

References

(1) Tamm, L. K.; McConnell, H. M. Supported Phospholipid Bilayers. *Biophys. J.* **1985**, *47*, 105–114.

(2) McConnell, H. M. Structures and Transitions in Lipid Monolayers at the Air-Water Interface. *Annu. Rev. Phys. Chern* **1991**, *42*, 95.

(3) Khattari, Z.; Sayyed, M. I.; Qashou, S. I.; Fasfous, I.; Al-Abdullah, T.; Maghrabi, M. Interfacial Behavior of Myristic Acid in Mixtures with DMPC and Cholesterol. *Chem. Phys.* **2017**, *490*, 106–114. https://doi.org/10.1016/j.chemphys.2017.04.012.

(4) Serro, A. P.; Galante, R.; Kozica, A.; Paradiso, P.; da Silva, A. M. P. S. G.; Luzyanin, K. V.; Fernandes, A. C.; Saramago, B. Effect of Tetracaine on DMPC and DMPC + Cholesterol Biomembrane Models: Liposomes and Monolayers. *Colloids Surf. B Biointerfaces* **2014**, *116*, 63–71. https://doi.org/10.1016/j.colsurfb.2013.12.042.

(5) Matyszewska, D.; Tappura, K.; Orädd, G.; Bilewicz, R. Influence of Perfluorinated Compounds on the Properties of Model Lipid Membranes. *Journal of Physical Chemistry B* **2007**, *111* (33), 9908–9918. https://doi.org/10.1021/jp068874g.

(6) Matyszewska, D.; Jocek, A. The Effect of Acyl Chain Length and Saturation on the Interactions of Pirarubicin with Phosphatidylethanolamines in 2D Model Urothelial Cancer Cell Membranes. *J. Mol. Liq.* **2021**, *323*. https://doi.org/10.1016/j.molliq.2020.114633.

(7) Luviano, A. S.; Campos-Terán, J.; Langevin, D.; Castillo, R.; Espinosa, G. Mechanical Properties of DPPC-POPE Mixed Langmuir Monolayers. *Langmuir* **2019**, *35* (51), 16734–16744. https://doi.org/10.1021/acs.langmuir.9b02995.

(8) Miñones, J.; Conde, O.; Dynarowicz-Łątka, P.; Casas, M. Penetration of Amphotericin B into DOPC Monolayers Containing Sterols of Cellular Membranes. *Colloids Surf. A Physicochem. Eng. Asp.* **2005**, *270–271* (1–3), 129–137. https://doi.org/10.1016/j.colsurfa.2005.05.052.

(9) Qiao, L.; Ge, A.; Liang, Y.; Ye, S. Oxidative Degradation of the Monolayer of 1-Palmitoyl-2-Oleoyl-Sn-Glycero-3-Phosphocholine (POPC) in Low-Level Ozone. *Journal of Physical Chemistry B* **2015**, *119* (44), 14188–14199. https://doi.org/10.1021/acs.jpcb.5b08985.

(10) Domènech, Ò.; Sanz, F.; Montero, M. T.; Hernández-Borrell, J. Thermodynamic and Structural Study of the Main Phospholipid Components Comprising the Mitochondrial Inner Membrane. *Biochim. Biophys. Acta Biomembr.* **2006**, *1758* (2), 213–221. https://doi.org/10.1016/j.bbamem.2006.02.008.

(11) Davies J.T.; Rideal, E. K. Surface Potential at Liquid Interfaces. In *Modern Aspects of Electrochemistry*; Academic Press, 1963.

(12) Matyszewska, D.; Moczulska, S. Effect of PH on the Interactions of Doxorubicin with Charged Lipid Monolayers Containing 1,2-Dimyristoyl-Sn-Glycero-3-Phospho-L-Serine - An Important

Component of Cancer Cell Membranes. *Electrochim. Acta* **2018**, *280*, 229–237. https://doi.org/10.1016/j.electacta.2018.05.119.

(13) Martin, A. L.; Jemmett, P. N.; Howitt, T.; Wood, M. H.; Burley, A. W.; Cox, L. R.; Dafforn, T. R.; Welbourn, R. J. L.; Campana, M.; Skoda, M. W. A.; Thompson, J. J.; Hussain, H.; Rawle, J. L.; Carlà, F.; Nicklin, C. L.; Arnold, T.; Horswell, S. L. Effect of Anionic Lipids on Mammalian Plasma Cell Membrane Properties. *Langmuir* **2022**. https://doi.org/10.1021/acs.langmuir.2c03161.

(14) Zaborowska, M.; Broniatowski, M.; Wydro, P.; Matyszewska, D.; Bilewicz, R. Structural Modifications of Lipid Membranes Exposed to Statins – Langmuir Monolayer and PM-IRRAS Study. *J. Mol. Liq.* **2020**, *313*, 1–11. https://doi.org/10.1016/j.molliq.2020.113570.

(15) De Almeida, R. F. M.; Fedorov, A.; Prieto, M. Sphingomyelin/Phosphatidylcholine/Cholesterol Phase Diagram: Boundaries and Composition of Lipid Rafts. *Biophys. J.* **2003**, *85* (4), 2406–2416. https://doi.org/10.1016/S0006-3495(03)74664-5.

(16) Vaknin, D.; Kelley, M. S.; Ocko, B. M. Sphingomyelin at the Air-Water Interface. *Journal of Chemical Physics* **2001**, *115* (16), 7697–7704. https://doi.org/10.1063/1.1406501.

(17) Rujoi, M.; Borchman, D.; DuPré, D. B.; Cecilia Yappert, M. Interactions of Ca2+ with Sphingomyelin and Dihydrosphingomyelin. *Biophys. J.* **2002**, *82* (6), 3096–3104. https://doi.org/10.1016/S0006-3495(02)75651-8.

(18) Villalain, J.; Ortiz, A.; Gomez-Fernandez, J. C. Molecular Interactions between Sphingomyelin and Phosphatidylcholine in Phospholipid Vesicles. *Biochim. Biophys. Acta* **1988**, *55*, 55–62. https://doi.org/10.1016/0005-2736(88)90213-1.

(19) Zaborowska-Mazurkiewicz, M.; Matyszewska, D. Lipid Envelopes of Influenza A and SARS-CoV-2 Virus – Physicochemical Description of 2D and 3D Models. *J. Mol. Liq.* **2025**, *423*. https://doi.org/10.1016/j.molliq.2025.126977.

(20) Santamaria, A.; Carrascosa-Tejedor, J.; Guzmán, E.; Zaccai, N. R.; Maestro, A. Unravelling the Orientation of the Inositol-Biphosphate Ring and Its Dependence on Phosphatidylinositol 4,5-Bisphosphate Cluster Formation in Model Membranes. *J. Colloid Interface Sci.* **2023**, *629*, 785–795. https://doi.org/10.1016/j.jcis.2022.09.095.
